# Antagonist activation exercises elicit similar post-activation performance enhancement as agonist activities on throwing performance

**DOI:** 10.1186/s13102-023-00657-9

**Published:** 2023-03-27

**Authors:** A. Pisz, D. Blazek, R. Jebavy, D. Kolinger, M. Wilk, M. Krzysztofik, P. Stastny

**Affiliations:** 1 Department of Sports Games, Faculty of Physical Education and Sport, Charles University, Prague, Czech Republic; 2 Athletic Department, Faculty of Physical Education and Sport, Charles University, Prague, Czech Republic; 3 Institute of Sport Sciences, Jerzy Kukuczka Academy of Physical Education in Katowice, Katowice, Poland

**Keywords:** Post-activation potentiation, Bench press, Push-up, Female athletes, Softball

## Abstract

**Background:**

This study aimed to determine the acute effect of agonist and antagonist conditioning activities (CA) on medicine ball throw performance among female softball players.

**Methods:**

Thirteen national-level female softball players (age 22.2 ± 3.1 years; body mass 68.3 ± 11.3 kg; softball experience 7.3 ± 2.4 years) performed 3 medicine ball chest throws before conditioning activity (CA) and after CA respectively in 3rd, 6th, and 9th minute. CA was the bench press and bent-over barbell row with 2 sets of 4 repetitions at 60% and 80% of one-repetition maximum, and 2 sets of 4 repetition bodyweight push up.

**Results:**

Two-way ANOVA revealed an increase in throwing distance (*p* < 0.001) after bent over barbell row and push-up exercise, and an increase in throwing speed (*p* < 0.001) after bench press and push-up. All performance increases were in moderate effect size (Cohen d 0.33–0.41), and no differences were found between the experimental CA.

**Conclusions:**

We conclude that upper body throwing performance is similar after antagonist exercise and agonist CA, both agonist and antagonist CA increase muscle power. In the resistance training practice, we recommend the interchange of agonist and antagonist CA using bodyweight push-up or submaximal intensity (80% of 1RM) bench press and bent over barbell row to succeed post-activation performance enhancement in upper limbs.

**Supplementary Information:**

The online version contains supplementary material available at 10.1186/s13102-023-00657-9.

## Background

Besides anthropometric characteristics and throwing technique, explosive strength is crucial for throwing in overhead athletes. Upper limb muscle power is an essential ability also in softball, where throwing performance is highly influenced by the rate of power development [1]. Therefore, the softball players and other overhead athletes are using advanced training methods like post-activation performance enhancement (PAPE) in long-term training programs. PAPE is an efficient method for an acute increase in explosive strength [2–6], which requires a precise selection of conditioning activity (CA) [7, 8]. Although PAPE is based on post-activation potential which increases the sensitivity of actomyosin filaments to Ca^2+^, enhances recruitment of higher-order motor units, and changes in pennation angle [9, 10], the exercise selection is one of the key factors to modulate the magnitude of PAPE [11].

It has been clearly shown that stronger individuals manage to reach greater post-activation responses and express it earlier than weaker counterparts [12]. However, to evoke the PAPE phenomenon, there needs to be a maintained balance between fatigue and potentiation, where the fatigue-potentiation response depends highly on activation exercise selection, load, and volume [10, 13]. In resistance training methods, it has been shown that antagonist training can reduce exercise fatigue, which increases exercise volume and intensity with concurrent shortening of rest intervals between two exercises [14, 15]. Intermuscular agonist and antagonist coordination are elementary adaptations to resistance training that increase strength and torque [16]. This is achieved by the neural strategy of enhanced reciprocal inhibition of antagonist musculature [17], where varied resistance and concentric antagonist speed of contractions affect subsequent concentric agonist effort. For instance, if contraction of the flexor muscle stimulates Golgi tendon organs that depolarize the Ib axon and fire nerve impulses that are disseminated to the spinal cord. This subsequently leads to a reduction of the flexor muscle tension associated with the extensor muscle spindles depolarization, thus leading to a more efficient contraction of the extensor [18]. This neural reciprocal effect may be the reason, why antagonist training can cumulate exercise volume or intensity and might be a potential aggregator of successful PAPE.

Most PAPE research focuses on agonist CA activity with similar biomechanical movement patterns [19, 20], which must balance between performance enhancement and possible fatigue [20]. Fatigue is the most evident effect of the contraction history, manifested by the incapability of the muscle to develop the desired level of force [21], and the fatigue can interchange the PAPE after CA. Therefore, measured response of muscular work after CA requires a net balance between processes that result in fatigue and potentiation [9], representing positive or negative performance responses to CA. In this fatigue-PAPE counterbalance, the antagonist CA might play a positive role, since the antagonist muscle might avoid metabolic fatigue of agonist groups and provide reciprocal inhibition activation. This has been reflected in setting up the PAPE rest interval, where short (5 min) [22], moderate (8–12 min) [23], and extensive (18.5 min) [24] recovery durations may elicit PAP. Although there is a finding that most athletes potentiate 6 min after CA, the optimal rest interval should be found by trial and failure approach [1].

Most of the current literature, including ballistic movements for improving upper body explosive power, has focused on male athletes [2, 5, 19, 25]. Only a few existing studies focus on its effect on female professionals [26–28]. Female athletes exhibit different muscle activity during bench press exercises than men [29], and therefore same conditioning might bring different results. It has been shown that 86% of women handball players are PAPE responders in throwing velocity to variable resistance intra-repetition method, and 93% of them positively respond to isometric CA [6]. In the exercise selection studies, the bench press (BP) CA increased throw distance in women's shot put [26], and maximal rowing exercise increased rowing speed [30] with the same PAPE effect for males and females rowers. Thus, the agonist PAPE has been shown in women athletes while the antagonist CA effect remains unknown.

The effect of antagonist muscle loading might be beneficial for the PAPE effect, which requires high motor unit pre-activation with low exhaustion of prime movers for performing an exercise. There is limited research on PAPE exercise selection in professional female athletes, and antagonist muscle activation effect, which has high practical use potential. Therefore, the study aimed to determine the acute effect of agonist and antagonist CA on medicine ball throw performance among female softball players. We hypothesize that submaximal antagonist CA should have a higher potentiation effect on power performance than agonists exercises at the same submaximal intensity.

## Methods

### Experimental approach to the problem

A randomized crossover and counterbalanced design were used to compare the effect of 80% submaximal bent over barbell row as antagonist CA against agonist bench press and control condition of push-up CA on ball throwing distance. The research has been run according to CONSORT guidelines (Additional file 1). Before launching 3 of the main experiment trials, subjects participated in 10 weeks of resistance training program to be familiarized with testing protocols, improve chosen exercises’ techniques, and increase strength. Moreover, during the last week of familiarisation, subjects performed 3 medicine ball throws (as used during the experiment) after each training visit to familiarize themselves with the throwing technique.

**Fig. 1:**
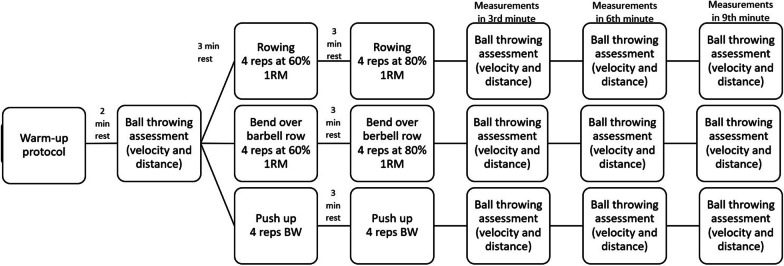
Flow chart of experimental protocol and sessions

There were three experimental sessions 48 h apart, where CA was included in randomized order for individuals (Fig. 1). After standardized warm-up subjects completed 3 experimental trials involving a baseline ball throwing with a 2 kg medicine ball followed by a CA. The push-up CA was performed in 2 sets of 4 repetitions with 3 min of rest between sets. Plyometric and isometric push-up exercises used as CA significantly improved shot put performance among female throwers [31], to adapt this exercise to the strength of the subjects it has been decided to perform push-ups as described below. The bent-over barbell row (ROW) and bench press (BP) were performed in 2 sets of 4 repetitions with respectively 60% and 80% one-repetition maximum (1RM) with 3 min rest between sets [32]. This intensity was chosen because both stronger and weaker individuals respond to CA better when stimuli are higher. Retesting of ball throwing was measured 3 times in total respectively after 3, 6, and 9 min. The load of 80% 1RM was chosen because it is enough to awake PAPE reaction with decreasing possible fatigue and harmful effects on results [1]. Recovery time was based on prior studies which show that gender and strength level influence rest time [3, 6, 11].

### Subjects

Power calculations (performed in G*Power 3.1.9.4) indicated that the minimum sample size of 12 participants would be required to detect an effect size of 0.4, collected from the average effect size reported in the Wilson metanalysis [21] (repeated measures, within-between interactions ANOVA power = 0.8, alpha = 0.05, correlation among rep measures = 0.8, number of groups = 3, number of measurements = 2. Thirteen (n = 13) professional Czech softball players from National Team and 1^st^ division Clubs volunteered in study (mean ± SD: age 22.2 ± 3.1 years; height 169 ± 4.5 cm; body mass (BM) 68.3 ± 11.3 kg; bench press 1RM 40.5 ± 6.8 kg; BP 1RM/BM 0.6 ± 0.1; bent over barbell row 1RM 39.5 ± 6.7 kg; ROW 1RM/BM 0.7 ± 0.1; softball experience 7.3 ± 2.4 years). Subjects were recruited on the basis that they were healthy, injury-free, and engaged in a resistance-training program for the last 10 weeks. They were able to perform bent-over barbell row and bench press with proper technique as assessed by certified strength and conditioning coach.

### Experimental procedures

Experimental trials were separated by 48 h from each other (Fig. 1). Moreover, subjects were instructed to avoid upper body workouts during the time of measurements. In the beginning, participants started with a standardized warm-up protocol consisting of 5 min of running on a treadmill with a constant speed of 6 km/h followed by dynamic stretching with an emphasis on stretching the chest musculature. Two minutes after warm-up, subjects performed ball throw as pre-measurement, and 3 min after pre-measurements, participants in a randomized order completed CA of either bent over barbell row, bench press, or push-up. The project coordinator assigns participants to groups using block randomization, random mixed block sizes were used so that participants could not predict the upcoming CA. After PAP activation subjects rested for 3 min before starting POST measurements.

### Conditioning exercises and one-repetition maximum measurements

#### 1 RM measurements

1RM of bench press and bent over barbell row was measured at the end of 10 weeks of resistance training familiarisation. Before measuring 1RM, all participants underwent a standardized warm-up consisting of 5 min of running on a treadmill with a constant speed of 6 km/h followed by dynamic stretching with an emphasis on stretching the chest musculature. All subjects started the exercise with 8 repetitions at 50% 1RM measured during the preparatory 10 weeks resistance training period. Then subjects performed 4 repetitions at 70% 1RM and 3 repetitions at 80% 1RM, respectively. After the final warm-up, subjects began lifting 1RM with maintaining proper technique and a full range of motion with the weight starting from 5 kg added to the previous 1RM. If an attempt was successful, 2.5 kg were added with rest intervals between each attempt for 3 min until proper 1RM was reached. Bent over barbell row 1RM was measured using the same warm-up and measurement protocol as during bench press.

#### Bench press technique

During a bench press, subjects were instructed to lay prone on the bench with the leg resting on the floor where the knee was positioned at a 90º angle. The grip was pronated with hands spaced in the distance between each other of 1.5 widths of the shoulder. Subjects were required to control the bar’s descent until the chest was touched approximately 3 cm above to xiphoid process and, without pause, push it. The cadence of the move was 2 s down and voluntary tempo up in the concentric phase controlled by the coach.

#### Bent over barbell row technique

**Fig. 2:**
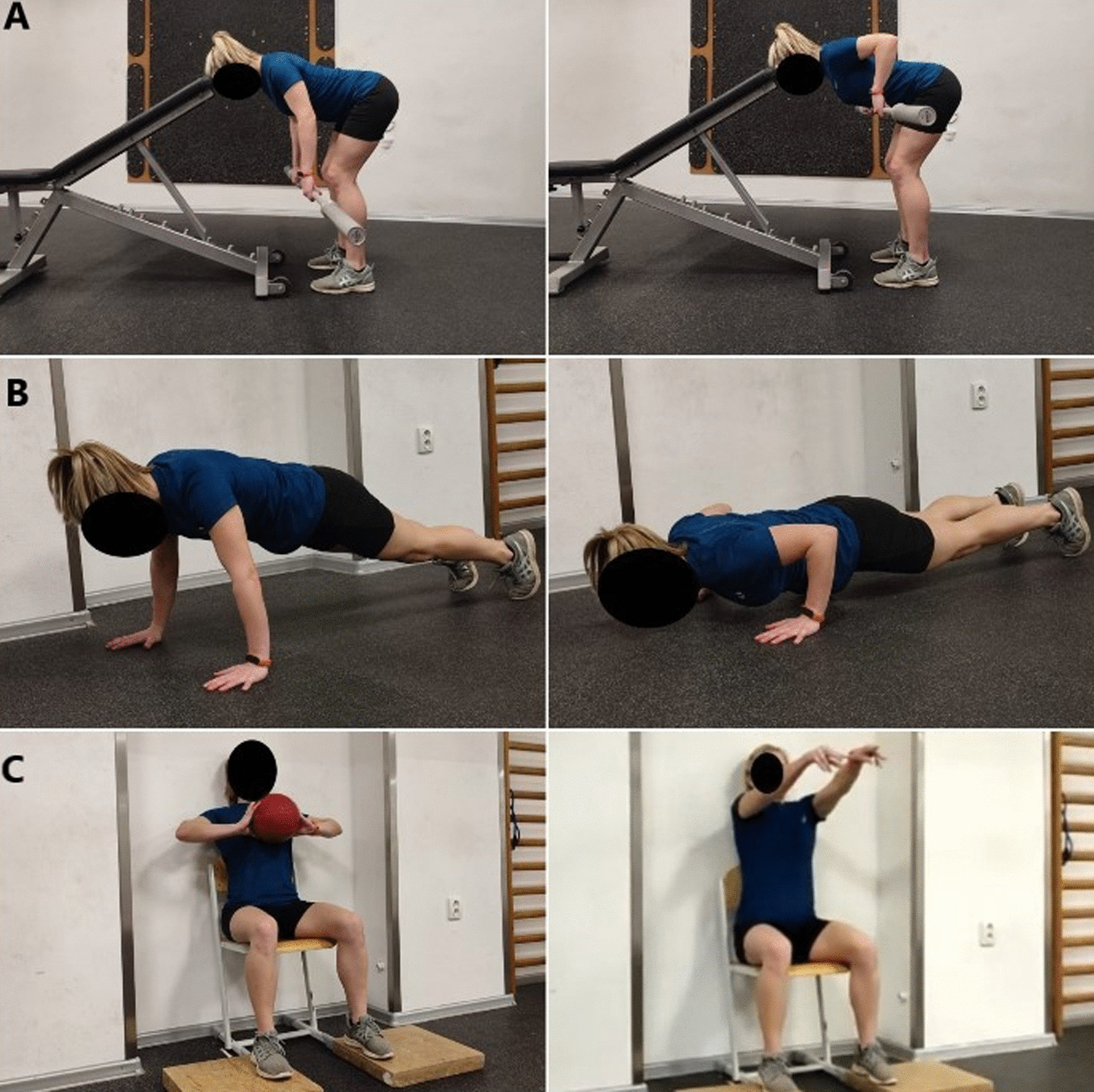
Pictures of conditioning activity and ball throwing positions. A = bent over row, B = push up, C = ball throw

The bent-over barbell row position required the head to lean on a bench with high adjusted to each person to maintain 90 degrees of flexion in the hip with knees slightly bent (Fig. 2). Participants were instructed to pull the bar at the height of the bottom of the sternum. The grip position was the width of the shoulders. The cadence of the move was 1 s up and 2 s down.

#### Push up technique

The push-up (PU) exercise was performed with wide hands positioned on the floor. It was completed from a plank position with the body remaining straight from the head to the heels. The starting position was with the placement of hands on the shoulder line with fingers pointing forward. When the view from the side, hands fell directly below the shoulders. From this position, subjects were instructed to lower the body with elbows directed to the sides until their chest almost touched the floor. After reaching that point subject changed direction without pausing. The cadence of the move was maintained 2 s down and voluntary tempo in the concentric phase.

### Medicine ball chest throw performance

The medicine ball throwing test used during this measurement is recommended by Harasin et al. [33] to measure maximum throwing performance. The test was performed in a sitting position where both legs were placed on the ground with 90º flexion in the knee. During each throw head and shoulders had to be in touch with the wall, and the trunk had to be in touch with a chair. The 2 kg medicine ball (circumference 65 cm) was used for throwing as participants practiced this weight before. Each participant was instructed to throw the ball with maximal effort and possibly furthest. Throws started from the chest position with elbows abducted from the trunk (Fig. 2). Measurement tape was placed on the ground to assess throw distance by an assistant with a distance meter. Each time ball had been thrown 3 times in the row without rest, the ball was passed to the participant immediately after a throw by an assistant. In front of the throwing position at the distance of 6 m was a standing assistant holding the radar (The Stalker ATS II, Version 5.0.2.1, Applied Concepts, Dallas, TX, USA) evaluating throwing speed (m/s). The best of 3 throws from each protocol was used to make statistical comparisons.

### Statistic

All statistic has been performed using STATISTICA software (TIBCO, PaloAlto, CA, USA), at alfa level 0.05. The normality was calculated by the Shapiro–Wilk test. One-way ANOVA was used to check the differences between initial throwing distance and maximum speed before the activation exercises. The Chi-square test was used to find whether there are differences in frequencies of responders, no-responders, and negative responders to CA. ANOVA for repeated measurements was used to calculate the throwing distance and maximum speed differences between time-series for pre and all post-measurements (repeated factor; pre-post 3 min, pre-post 6 min, pre-post 9 min), and Cohen’s d effect size was calculated between pre and all post measurements.

Two-way ANOVA for the repeated measure was used to calculate the throwing distance and maximum speed differences between pre and the best post-measurements (repeated factor) and between the CA type (repeated pre-post measure x CA exercise), followed by the Fisher LSD post hoc test. Fisher LSD test was used since we compared three groups for within-subject effect, to avoid type II errors [34]. The effect size (partial eta squared – η^2^) of each test was calculated for repeated measure factor and classified according to Larson-Hall [35] and Cohen [36], where η^2^: 0.02, 0.13, 0.26 were considered as small, moderate, and large effects respectively. The effect of the activation exercise was calculated by Cohen’s d effect size considering 0.2, 0,5, and 0,8 as small, medium, and large effect sizes, respectively.

## Results

**Fig. 3:**
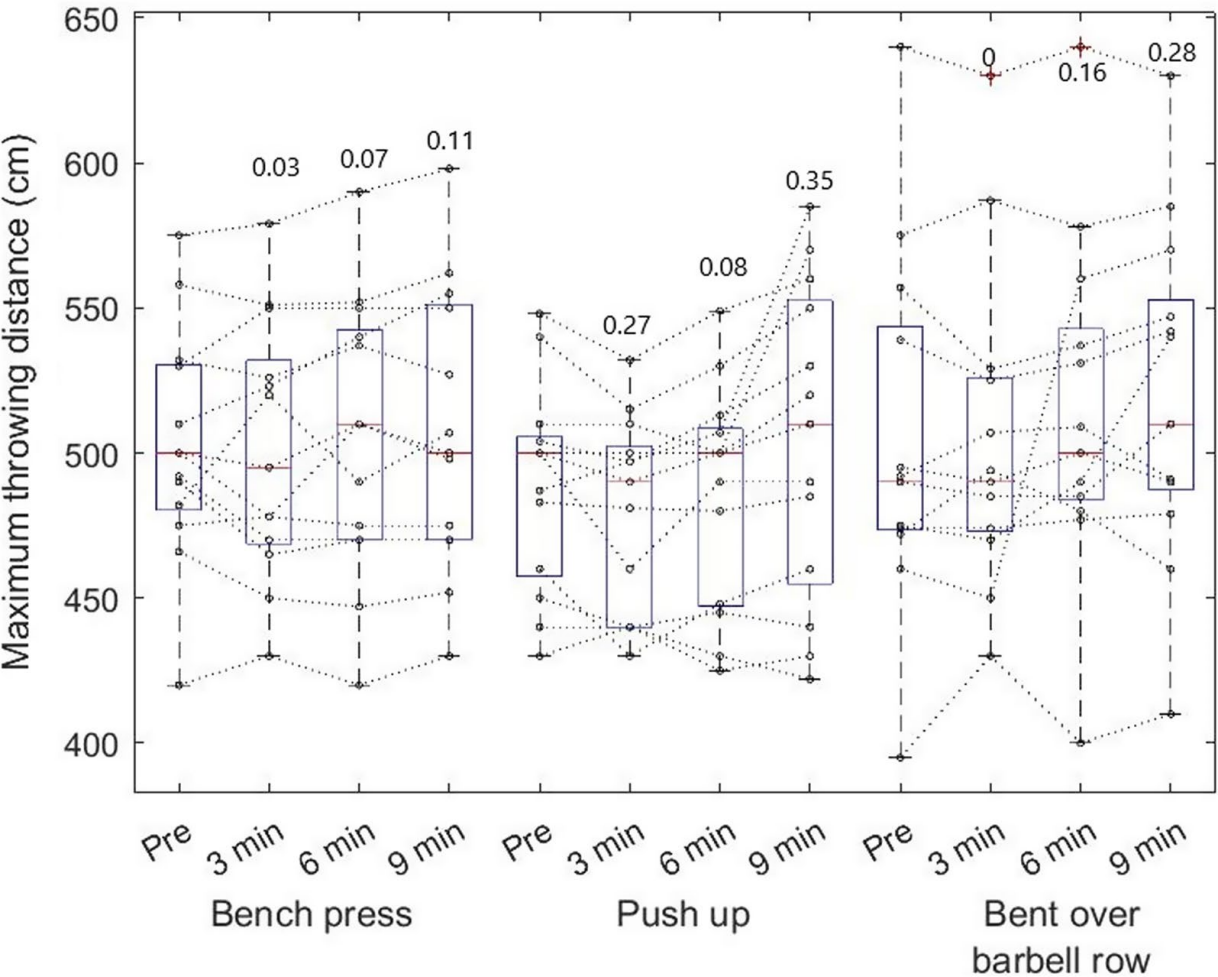
Individual throwing performance among softball players by maximum throwing distance (cm) during pre-measurements and time series of 3, 6, and 9 min after CA. Boxplots are expressed in median and the bottom and top edges of the box indicate the 25th and 75th percentiles, respectively. The numbers above the boxplot are Cohen's d comparing pre and particular minutes

**Fig. 4:**
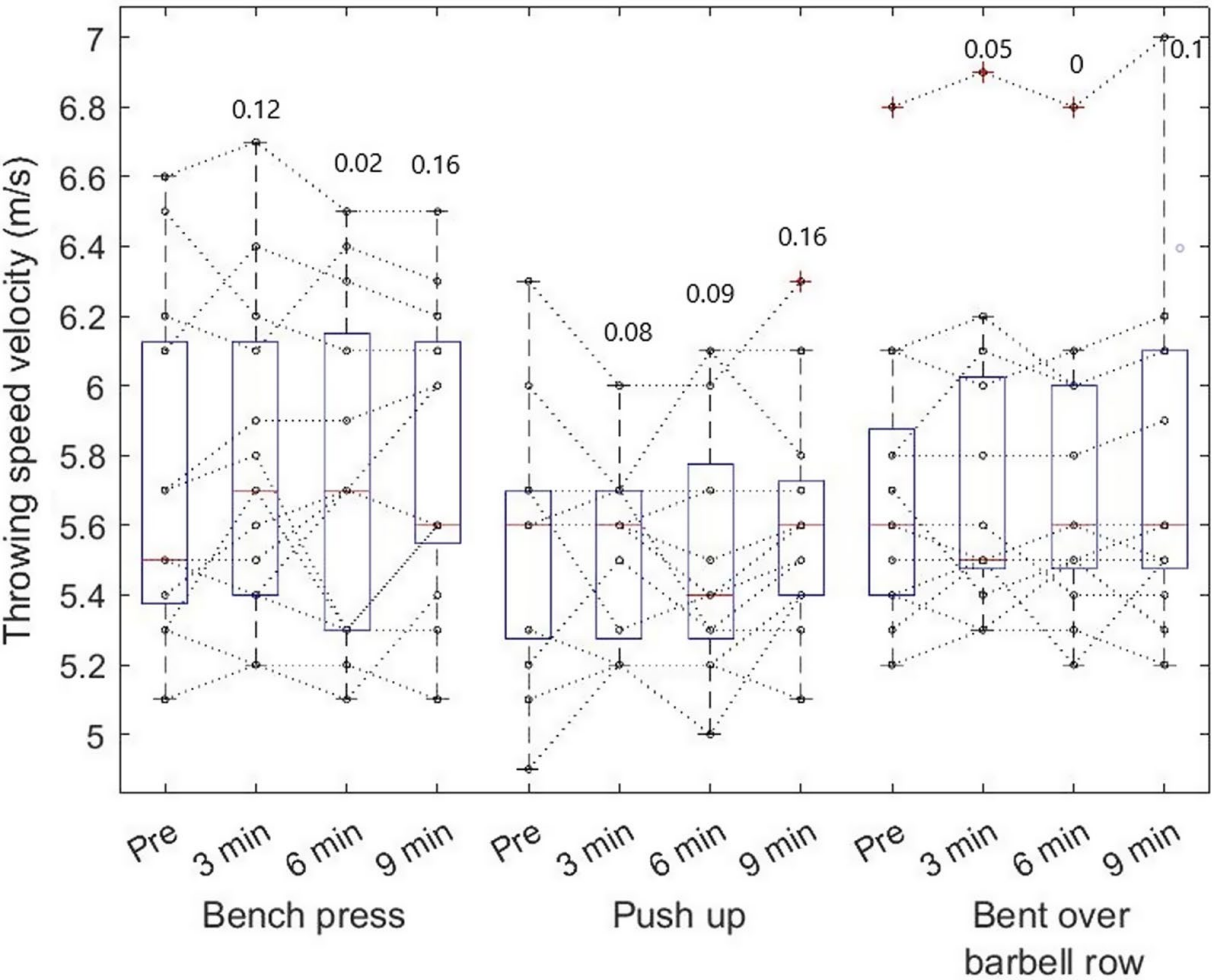
Individual throwing performance among softball players by maximum throwing distance (cm) during pre-measurements and in time series 3, 6, and 9th minute after CA. Boxplots are expressed in median and the bottom and top edges of the box indicate the 25th and 75th percentiles, respectively. The numbers above the boxplot are Cohen's D comparing pre and particular minutes

The data normality was not disrupted (Table 1, Additional file 2) and the two-way mixed absolute agreement intraclass coefficient in pre-values was 0.82 at CI 0.562 – 0.941. The initial values (Table 1) between all three activation exercises during pre-test were similar in distance (F_2, 24_ = 1.1, *p* = 0.35) and maximum speed (F_2, 24_ = 2.1, *p* = 0.13). The frequency of positive, no, and negative responders was the same for each CA (Table 2), with a maximum of 3 negative responders for CA. ANOVA for repeated measure did not show any statistical differences between time series of pre and 3, 6, and 9 min post measurements (Figs. 3, 4). However, there was a moderate effect size between pre and post-9 min after push-up and bent over barbel raw in throwing distance (Fig. 3). On the other hand, there was a moderate performance decrease in 3 min post measurement after push-up.

**Table 1:** The effect size and data distribution of maximal ball throw distance and speed before and after conditioning activities respectively

	Conditioning activity	Mean ± SD		Within effect size (Cohen's d)	Shapiro–Wilk		95% CI	
		Pre-activation	Post-activation		Pre-activation	Post-activation	Lower Bound	Upper Bound
Distance (cm)	Bench press	502.31 ± 40.4	510.92 ± 49.1	0.19	0.97	0.97	477.88	526.74
	Bent over barbell row	504.15 ± 61.1	527.54 ± 52.8	0.41	0.92	0.97	467.24	541.06
	Push up	488.62 ± 35.8	506.54 ± 51.0	0.37	0.95	0.93	466.97	510.26
Maximum speed (m/s)	Bench press	5.72 ± 0.5	5.91 ± 0.4	0.41	0.90	0.97	5.43	6.01
	Bent over barbell row	5.72 ± 0.4	5.82 ± 0.5	0.23	0.89	0.83	5.46	5.97
	Push up	5.56 ± 0.4	5.68 ± 0.3	0.33	0.95	0.92	5.34	5.79

SD, standard deviation; CI, confidence interval

**Table 2:** The number of responders to different conditioning activities

Condition activity	Responders (n)		Non-responders (n)		Negative responders (n)	
	Distance	Speed	Distance	Speed	Distance	Speed
Bench press	10	11	0	0	3	2
Bent over	11	10	1	1	1	2
Push up	9	7	2	4	2	2

**Fig. 5:**
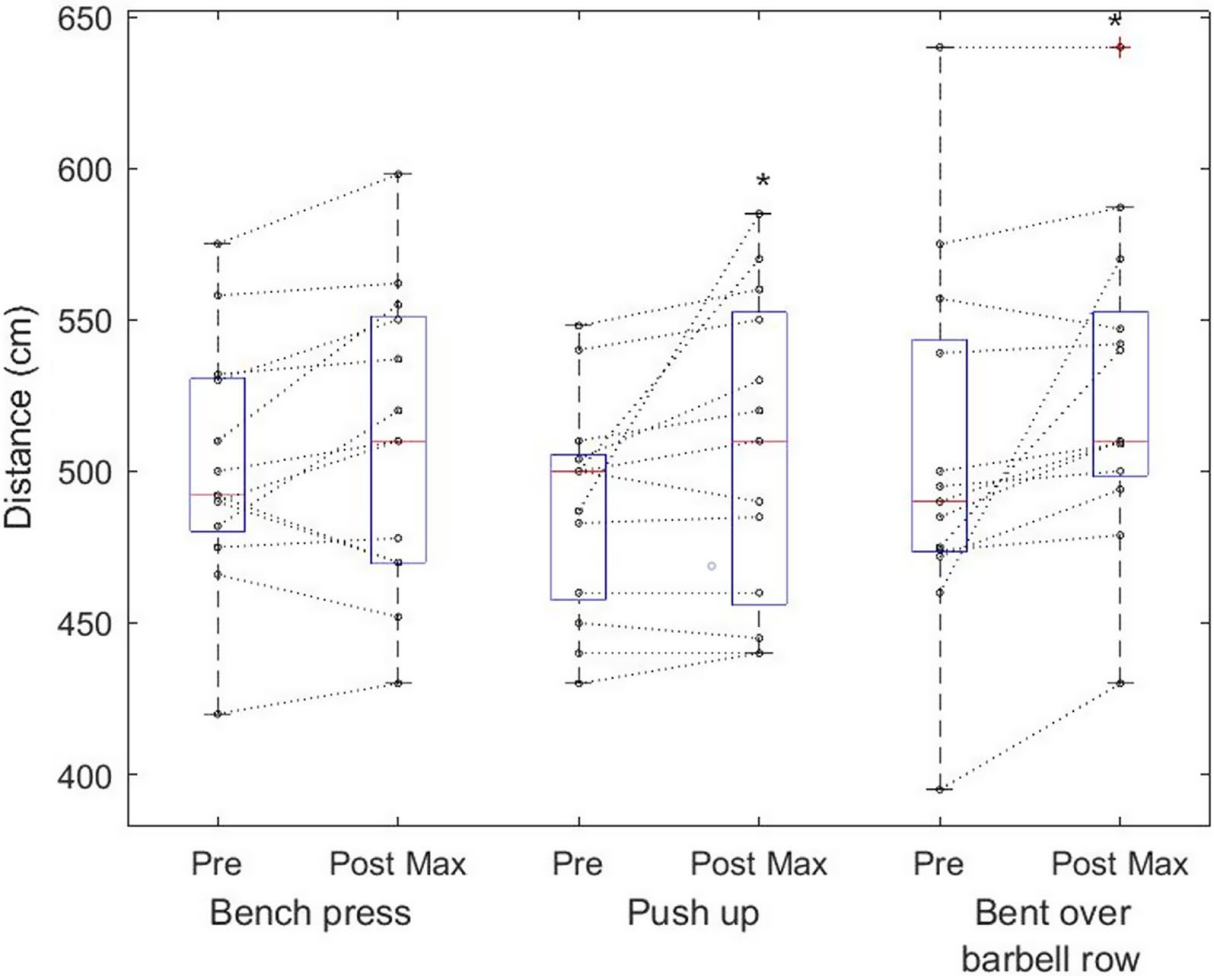
Individual throwing performance among softball players by maximum throwing distance (cm). Boxplots are expressed in median and the bottom and top edges of the box indicate the 25th and 75th percentiles, respectively. * denote significant performance increase by ANOVA and Fisher LSD post hoc test

**Fig. 6:**
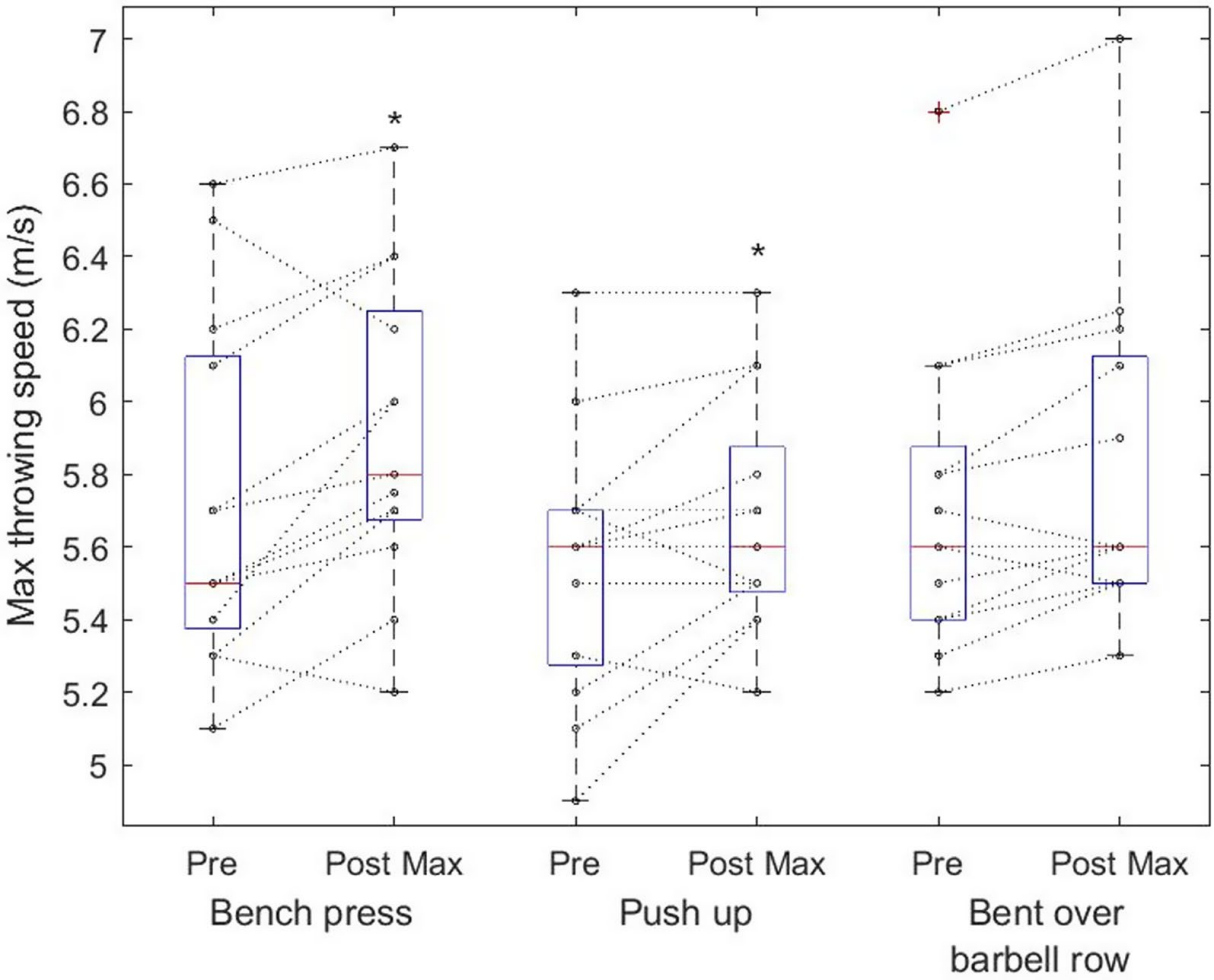
Individual throwing performance among softball players by maximum throwing speed (m/s) during pre-measurements and in respectively 3, 6, and 9th minutes after CA. Boxplots are expressed in median and the bottom and top edges of the box indicate the 25th and 75th percentiles, respectively. * denote significant performance increase by ANOVA and Fisher LSD post hoc test

The two-way repeated measure ANOVA showed differences in throwing distance (F_1, 36_ = 13.5, *p* < 0,001, η^2^ = 0.27), whereas the post hoc test showed increased throwing distance after bent over row and push-ups (Fig. 5). Further differences were found in throwing speed (F_1, 36_ = 19.9, *p* < 0.001, η^2^ = 0.36), where the post hoc test showed increased speed after bench press and push-up (Fig. 6). There was no interaction between the CA´s.

## Discussion

Our hypothesis was based on the neuromuscular activation link between the antagonist’s muscles, which should have a higher potentiation effect on power performance than the agonist´s exercises at the same submaximal intensity. This hypothesis was not confirmed as we observed similar effects on all three types of conditioning activities. This finding agrees with the general approach that CA exercise selection modulates the performance effect [14], and antagonist activation allows similar or better performance enhancement.

In comparison to other studies targeting the effect of PAPE on female upper body performance [37–43], our results agree with Martinez-Garcia [6] and Evetovich [26], where female athletes showed significant improvement in throwing velocity and shot put throwing distance after using bench pressing as CA. Similar outcomes were obtained also for agonist rowing exercise used as CA in measurements reporting moderate to large increases in average power output (+ 2.5%), peak power output (+ 1.5%), and power output during a first stroke in the 10-s maximal rowing performance (+ 0.79%) [30]. Moreover, agonist-resisted ergometer rowing elicits an increase in mean rowing power after dynamic potentiating when compared to isometric potentiating and the control group [44]. Another similarity to our results is in push-up exercise, which was successfully used as CA among female athletes to boost shot put performance [31]. Specifically, using isometric push-ups improves shot put throwing distance with a significant effect of 3.59 ± 2.7% [31]. In the antagonist CA, we can compare our results only to the study of Bayazit [45], who concluded that bent over row applied before shooting in archery might be an effective method of improving scoring means, although this effect was not statistically significant. For this study we selected the bent-over barbell rowing based on previous experimental designs, and because it evokes large muscle activation symmetrically from the upper to lower back [46]. However, our study confirms the knowledge that agonist BP, agonist pus-up, and antagonist bent over row are possible appropriate CA´s for successful PAPE effect on medicine ball chest throw distance and speed performance among females after appropriate resistance training preparation. Therefore, it might be beneficial to interchange different types of conditioning to evoke a PAPE response.

One of the keys to positively improving body explosive power output is properly adjusted protocols for CA exercise [47, 48]. This study shows significant improvement in maximal throwing ball speed after 2 sets of 4 repetitions of BP and bent over barbell row, with respectively 60% and 80% 1RM for trained female softball players. The submaximal intensity was chosen because both stronger and weaker individuals respond to CA better when stimuli are higher [10, 49], however, the appropriate exercise volume must be adjusted because there is a significant effect of strength training experience on time under tension and number of repetitions [50]. In our athletes, the BP intensity most likely increased the recruitment of higher-order (type II) motor units [51]. However, it could be speculated that higher intensity might disrupt the throwing technique and accuracy due to the resting muscle twitch response in the agonist’s muscles.

The most uncertain loading parameter to elicit PAPE is the rest interval, where a previous study on male athletes reported 6 min as the most frequent optimal rest interval [1]. This is different from our finding, which suggests the biggest potentiation effect in the 9^th^ minute (Figs. 5 and 6). One of the explanations might be that women athletes require a longer break after exercise to balance fatigue and potentiation ratio [52]. Sale [53] concluded that the longer the interval between the end of the conditioning activity and the start of the performance, the greater the recovery from fatigue, but also the greater the breakdown of the PAP mechanism. Additionally, it can be speculated that serial testing (every 3 min) could in some cases promote a positive effect by reactivating PAP, in this case, it may have the effect of aggravating an already high level of fatigue [54]. Concerning these assumptions, it is possible to speculate that the participants in this study either required a longer post-exercise break to recover or that they compounded their activity after several trials.

PAPE has been widely used to improve the specific performance of athletes [55–57]. Since ball-throwing performance was improved after antagonist and agonist activation, there is a question whether it would work in other sports activities, such as combining upper and lower limb take-offs and spikes in volleyball [58, 59], which might be investigated in future research. Another investigation field should be modifications of the conditioning contraction or type of conditioning activity. Typically, concentric activation might be used to induce potentiation [60, 61], but previous research proved that using eccentric [62] and unilateral loading [63] as a conditioning activity evokes PAPE as well. In addition, the localization of potentiation has also been questioned recently with the major question of whether the effect occurs only in the muscles involved in the conditioning activity or if it is more global. In the study made by Bartolomei [64] a high intensity bench press showed a significant improvement in jump performance. This shows that PAPE can have a non-local effect, meaning that through complex upper-body resistance training, there is a potential to improve lower-body performance. This is another suggestion for future studies to assess if antagonist activation can also induce non-local PAPE and under what circumstances, and whether a change in the type of contraction in the conditioning activity can affect potentiation.

A potential study limitation is that only medicine ball chest throws were used to assess the effect of CA on upper body explosive performance. This method is often used for examination training effects [6, 42], as it allows constrained use of body segments [42]. On the other hand, the throwing position causes more stability and, therefore, less variability in throwing attempts [27, 43]. Moreover, the results might be affected by throwing ball weight and throwing technique [44]. Subsequently, there is a question for further research, what antagonist exercise intensity and volume are best for the highest PAPE effect? Another limitation is the intensity of push-ups which might differ among participants and missing physiological mechanisms examination which could explain obtained results. Additionally throwing distance measuring accuracy was dependent on assistant evaluation which might be a subjective opinion and therefore might lead to minor differences.

## Conclusion

In the resistance training practice, we recommend the interchange of agonist and antagonist PAPE stimulation at push-up bodyweight or BP and bent over raw submaximal (80% of 1RM) intensity to succeed PAPE on upper limbs to avoid culminating fatigue. Our study presents a new finding that it is possible to use bent-over barbell row as CA to improve throwing distance, or to alternatively use push-ups which were recommended previously. When aiming to improve throwing speed, the bench press and push-up seems to be the most appropriate exercise for CA. In contrast to men's studies, the greatest improvement in performance among female softball players is using 9-min rest intervals after a CA.

## Supplementary Information

**Additional file 1**. CONSORT checklist.

**Additional file 2**. Dataset.

## Data Availability

All data are available in the Additional file 2, the data table.

## Abbreviations

CA: Conditioning activity
PAPE: Post-activation potentiation enhancement
BP: Bench press
1RM: One repetition maximum
PU: Push up
ROW: Bent-over barbell row
